# The science of bioelectrical impedance-derived phase angle: insights from body composition in youth

**DOI:** 10.1007/s11154-025-09964-7

**Published:** 2025-04-10

**Authors:** Gil B. Rosa, Henry C. Lukaski, Luís B. Sardinha

**Affiliations:** 1 Exercise and Health Laboratory, Faculdade de Motricidade Humana, CIPER, Universidade de Lisboa, Cruz-Quebrada, Portugal; 2 Department of Kinesiology and Public Health Education, Hyslop Sports Center, University of North Dakota, Grand Forks, ND USA

**Keywords:** Bioimpedance, Capacitance, Disease, Electrical conductivity, Health biomarker, Pediatric

## Abstract

Despite bioelectrical impedance analysis (BIA)-derived phase angle (PhA) being recognized as a global marker of health, reflecting both cellular integrity and fluid distribution, its biological determinants still need to be described in youth. This narrative review provides a comprehensive framework examining to what extent dielectric properties shaping PhA are influenced by qualitative and quantitative determinants at multiple levels of body composition in healthy and clinical pediatric populations. At the atomic-molecular level, water content, glycogen, lipids, and ionic concentrations are expected to influence PhA by affecting electrical conductivity and/or capacitance. While the increase in the absolute values of intracellular (ICW) and extracellular water (ECW) enhances electric conductivity, an increase in the relative portion of ECW is expected to reflect hydration imbalances with an impact on electrical pathways. At the cellular level, body cell mass is a key determinant of PhA, mainly due to the presence of skeletal muscle cells favoring conductive and capacitive properties. At the tissue level, skeletal muscle architecture and orientation strongly influence conductivity, while increases in skeletal muscle mass positively impact PhA by enhancing electric conductivity and capacitance. Beyond the theoretical insights presented in this review, careful interpretation of dielectric data remains crucial due to the lack of methodological standardization. Future research should prioritize validated reference methods, investigate longitudinal changes, integrate localized BIA, and explore additional BIA models to refine the interpretation of PhA.

## Introduction

Global research has moved towards identifying potential cellular integrity and function markers arising from the biophysical characteristics of both cells and biological tissues in response to exogenous sources of alternating electric current flow techniques (e.g., bioelectrical impedance analysis, BIA). These techniques have been used to monitor the conduction of applied alternating current, measuring both the electric stimulation through cells and tissues and the voltage difference detected between the current-introducing and voltage-sensing electrodes.

Considering the ratio between the maximum voltage drop and current level, measuring the total opposition that a circuit presents to an alternating applied current– i.e., electrical impedance (Z) [1] is possible. In the context of alternating current circuits, Z is a measurable property determined from two biophysical parameters - resistance (R) and reactance (X) [1]. Whereas R refers to the extra and intracellular opposition that the human body presents to the flow of this electric current (i.e., inversely related to conductive properties), X represents the ability to temporarily store electric charge at the cellular level (i.e., capacitive properties). Due to the presence of capacitive elements within specific cellular organelles, such as cell membranes, which store energy in the form of an electric field, the corresponding X is typically represented as capacitive reactance (Xc). This raw BIA parameter is a function of capacitance and is inversely proportional to the signal frequency [2]. Depending on the relative contribution of both R and Xc of complex biological conductors, which rely on distinct dielectric properties (e.g., capacitivity and conductivity), it becomes possible to ascertain the degree to which the current leads or lags the voltage in an alternating circuit. In the human body, this phase shift is mostly determined by capacitive-related properties [1]. This delay is described as the phase angle (PhA, °), which is an angular index of the delay between current and voltage drop at the cell membrane and can be calculated by the arctangent of (Xc/R) × 180°/π [1].

Unlike R and Xc, which must be normalized to specific conductivity and relative permittivity (i.e., ability to permit storage of electric energy) for comparisons across individuals, PhA is gaining more interest for several reasons. Among these, two standout features are that PhA does not require adjustments for body size or empirical corrections [3] and that it does not change across various electric circuit models (i.e., series or parallel) [4]. As the ratio of Xc/R remains relatively stable across different body sizes, the body size normalization naturally cancels out, making PhA a more robust parameter to compare across different populations [3]. With the latest literature suggesting PhA as an index of extracellular-to-intracellular water ratio (ECW: ICW), body cell mass (BCM), and cellular integrity, this raw BIA parameter has been widely used as a non-invasive nutritional and health assessment tool in pediatric and adult populations.

To the present date, most evidence shows that higher levels of PhA are conceptually associated with increased BCM, which is mainly composed of muscle cells and directly linked to higher contents of ICW than ECW (i.e., improved cell membrane health) [5]. This suggests that higher values of PhA can be found, for instance, in populations with favorable body composition parameters, such as athletes or physically active and healthy individuals. On the other hand, lower PhA values are simultaneously attributable to reduced BCM, increased proportion adipose tissue, and expansion of ECW: ICW ratio [2], which, in turn, are related to reduced physical fitness, compromised health, and increased rates of hospitalization and mortality [6]. In sum, the available investigations have given a special focus to PhA, suggesting this raw BIA parameter as a relevant marker of physical fitness and a prognostic tool indicative of disease progression (e.g., sarcopenia and sarcopenic obesity), hospitalization risk, and all-cause mortality, particularly in children, older adults, and other specific populations (e.g., athletes and clinical patients) [7, 8].

When it comes to the determinants of PhA, most investigations solely explored the impact of water distribution (i.e., molecular-level), BCM (i.e., cellular-level), and tissues’ proportions [9, 10], not showing a complete and concurrent understanding of the biological significance of multiple body composition variables on PhA and other biophysical variables. According to Wang et al. [11], body composition can be organized in a five-level model compromising the atomic, molecular, cellular, tissue, and whole-body components, arranged from the simplest to the more complex structures. At the lower levels, atoms represent the fundamental building blocks of body tissues, while the molecules are derived directly from the arrangement and abundance of these atoms [11]. Since atoms and molecules are closely interconnected and represent complementary aspects of the same fundamental components, they are frequently discussed together. As we advance in complexity, the focus shifts to more functional systems, where cells represent the body’s functional units, tissues exert specific physiological roles (e.g., structural support, energy metabolism, and locomotion), while the whole-body level considers the integration of all lower levels into the complete organism. Because whole-body variables provide a macroscopic perspective of lower-level components, most evidence examining the determinants of PhA has primarily focused on these measures, often overlooking more simple components that may offer more specific and informative insights.

With most physiological and morphological changes occurring during growth reflecting age-dependent alterations in body composition [12–15] – e.g., molecular level: the proportion of total body water (TBW) relative to body mass decreases by approximately 20% from infancy to adulthood; cellular level: BCM is expected to also double its contribution to body mass from early childhood to adulthood (i.e., ~ 20% to 45–55%); tissue level: skeletal muscle mass (SMM) proportion increases from 20% at birth to over 40% in a healthy adult, significant changes in PhA can be anticipated in youth. In fact, this is the period when the most rapid changes in PhA occur, with this raw BIA parameter nearly duplicating from infancy to late adolescence in both sexes [16]. While a few body components, including fat mass, fat-free mass, stature, and body mass index, have been identified as relevant markers of PhA at an early age [17–19], most evidence still fails to provide a comprehensive understanding of which specific body composition factors definitively determine PhA variability.

Due to the theoretical gap in understanding how various components of the body, beyond those previously described, influence raw BIA variables, we aimed to provide a comprehensive framework conceptualized as a narrative review to identify the qualitative implications of multilevel body composition determinants on PhA. Additionally, we sought to describe how quantitative changes in body components, occurring during the sensible period of youth, can impact raw BIA variables, especially PhA. This is of most interest in youth, as a better understanding of the qualitative and quantitative determinants of PhA can enhance its reverse application – i.e., unlocking its full potential as an informative marker of body composition during this critical stage of growth and development.

This review will initially explore the multilevel determinants of PhA in youth by adopting a qualitative perspective emphasizing body composition determinants at the atomic-molecular, cellular, and tissue levels. Then, attention will be given to the implication of quantitative changes in these multilevel determinants, occurring in the sensible period of youth, on raw BIA variables. With a particular focus on health-compromised youth, the review will also address how raw BIA variables may vary according to disease type. Finally, relevant scientific remarks and future recommendations will be discussed to guide and enhance future investigations. In addition, the following aspects must be highlighted: (i) only the main determinants of electrical properties are highlighted, acknowledging the minor role of other variables (e.g., bone tissue); (ii) closely interrelated components across different levels are not discussed to avoid redundancy; (iii) the focus on lower levels provides a more detailed understanding of their influence on raw BIA measurements, often obscured in whole-body analysis; and (iv) given variations in raw BIA parameters due to device specifications, this review prioritizes investigations using tetrapolar, single-frequency (50 kHz) devices for standardized insights into PhA and body composition in youth.

ft.

## Multilevel body composition determinants of phase angle in youth – qualitative perspective

**Fig. 1:**
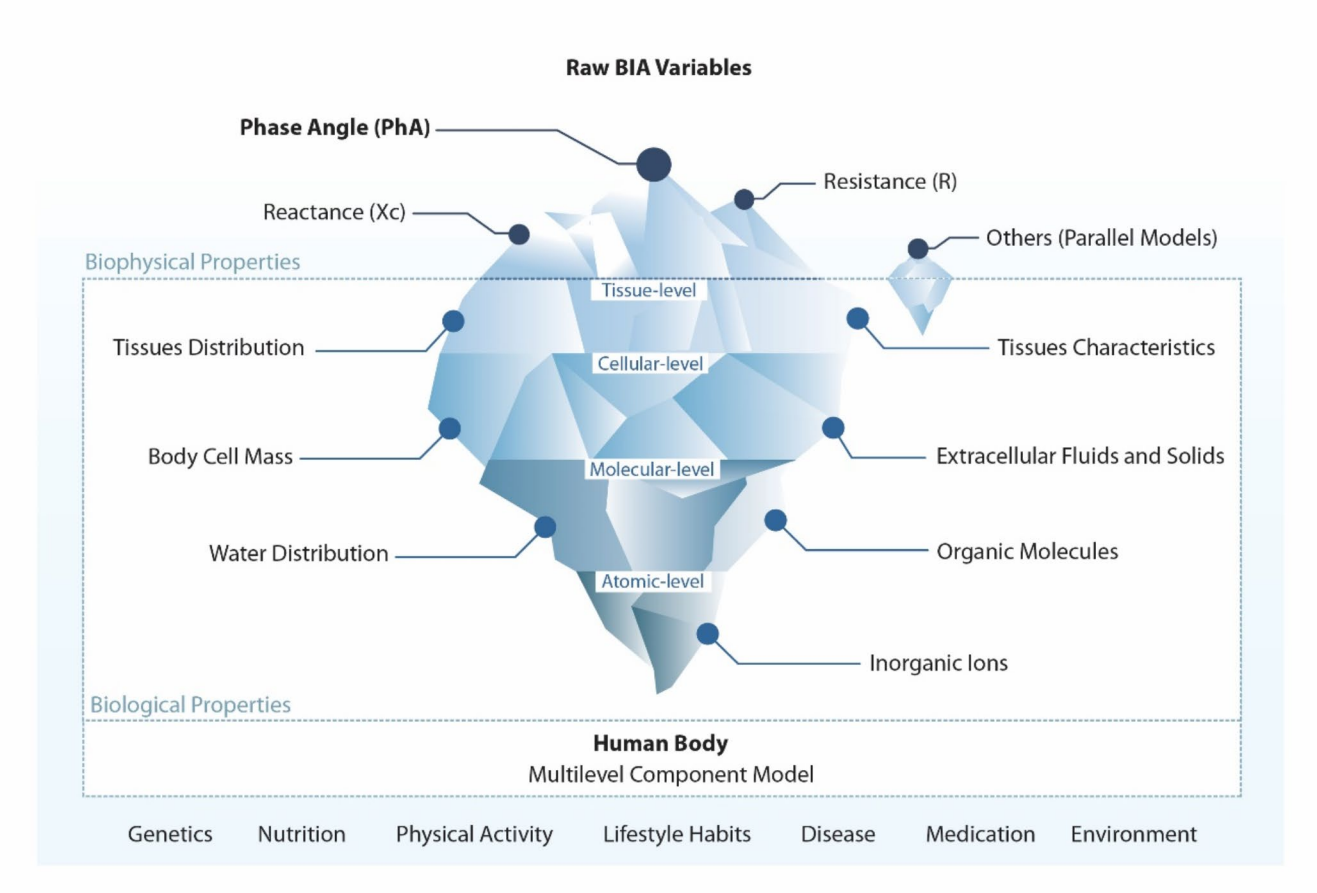
Iceberg model of bioelectrical impedance analysis: surface biophysical indicators and underlying biological properties

Since the beginning of the last century, investigations have suggested that raw BIA-derived parameters, particularly PhA, are determined by several factors directly influencing the overall characteristics of cells and the organization of structural tissues. By adopting a biophysical point of view, this review will describe how relevant atomic-molecular, cellular, and tissue-level determinants can influence the conductive and capacitive properties of the human body, which will further modulate PhA (Fig. 1).

### Atomic and molecular-level determinants

**Fig. 2:**
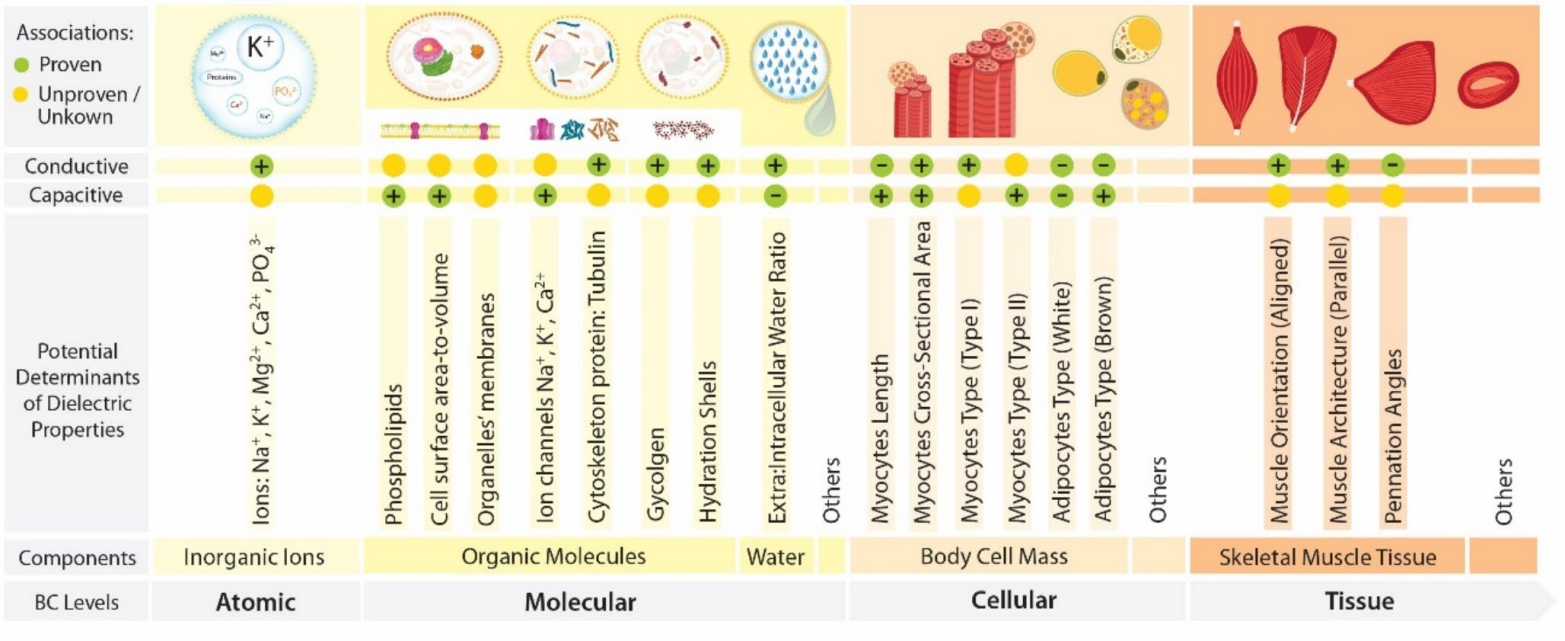
Potential implications of multilevel body composition components on dielectric properties in humans

When discussing micro-level determinants of raw BIA variables, we typically refer to distinct constituents implied in atomic-molecular processes that can influence the cell’s biophysical properties. Within this research field, relevant cell constituents include water, as well as other elements such as inorganic ions and organic molecules (Fig. 2, **Atomic - Molecular**).

#### Inorganic ions

Accounting for 70 to 80% of BCM, water is the most abundant molecule in cells [20]. In the human body, cellular water is not inherently pure. Instead, it contains multiple dissolved ions that are electrically charged and sustain electrical conductivity [20, 21]. This fundamental concept was first demonstrated by Rudolf Höber in 1910 through in vitro experiments showing that the electrolytic cytoplasm of compacted blood and muscle cells, which contained free ions within a plausible physiological range, is a relevant determinant of electric conductivity [21]. While hypothesizing that conductivity directly correlates with the concentration of ions dissolved in an aqueous solution, Meguid and colleagues demonstrated a direct association between Na^+^ concentration and conductivity using dialysis tubing [22]. Similar trends were documented in other research areas exploring water conductivity properties from various sources [23]. Higher conductivity rates were found in highly enriched water with ions but not in relatively pure types of water (e.g., distilled solutions), independently of the measurement methods (i.e., number of electrodes and frequency levels) [23].

While certain cellular processes supporting growth and bone mineralization (e.g., signal transduction and gene expression) [24], could suggest increased intracellular levels of specific electrolytes, particularly those directly involved in these processes (e.g., Ca²⁺ and PO₄³⁻), potentially favoring intracellular conductivity, there is still limited evidence to confirm this hypothesis. Instead, the available literature points towards similar concentrations of the most prevalent inorganic ions (e.g., Na⁺, K⁺, Mg²⁺) across all ages, particularly in healthy populations. Nevertheless, direct verification is still required, especially through experiments using in vivo methods (e.g., neutron activation analysis), to determine the real cytoplasmatic concentrations of inorganic ions and assess their relationship with BIA-derived parameters. Moreover, further evidence is required in youth to investigate the frequency-dependent nature of conductivity and its role in how atomic processes (e.g., drifting ions) influence BIA measurements. A major challenge relies on the fact that most available techniques provide only a systemic overview of inorganic ions without distinguishing between intracellular and extracellular compartments, whereas other physiological factors, such as the cell structure and internal temperature [25], may also influence the electrical path within the cell environment.

#### Organic molecules

##### Lipids

Lipids, such as fatty acids (FA), are the fundamental building blocks of the cell membrane and other cellular organelles, operating structural, metabolic, and signal transduction functions. While looking at the implications of lipids on electrical conductivity, Hodkin and Huxley [26] have early documented the bioelectrical phenomena when the electric current is carried through lipidic structures, such as the cell membrane. The organization of the cell membrane as a bilayer lipid membrane (BLM) enables the formation of an insulating barrier between the electrically conductive solutions of the intra- and extracellular mediums, with the potential to retain energy through the capacitive properties of the FA chains (i.e., the hydrophobic surface of each monolayer; capacitance of ~ 1 microfarad µF/cm^2^) [27].

Despite the rapid turnover of membrane lipids, the dietary FA composition can significantly influence membrane lipid structure and function. Previous evidence has shown that membrane-linked cellular processes contributing to energy metabolism remain relatively constant in response to saturated and monounsaturated FA diets but are significantly affected by polyunsaturated FA, such as n-6 and n-3 polyunsaturated FA [28]. A similar pattern has been observed in electrical capacitance, with this biophysical property being particularly sensitive to changes in membrane lipids. For instance, studies on NG108-15 cells demonstrated that dietary supplementation with polyunsaturated FA induced significant changes in membrane capacitance, reflecting alterations in membrane lipid dynamics [29]. More recently, in vivo experiments using whole-cell patch clamping to record cardiomyocyte electrical activity demonstrated that treatment with multiple FA enhances membrane capacitance, likely reflecting an increase in cell surface area [30].

When looking at lipidic structural characteristics in youth, although phospholipids composition assumes adult levels of most FA before the second year of life, some FA are expected to be higher in youth [31], potentially enhancing the capacitive properties of the cell membrane. For example, monounsaturated FA, such as oleic acid (i.e., 18:1 n-9 FA; most prevalent monounsaturated FA), are expected to peak in the erythrocyte membrane lipids during childhood and then gradually decrease until adulthood [31]. Considering the unique characteristics of this monounsaturated FA (i.e., kink structure), previous evidence has shown a close relationship between the content of this unsaturated FA in the membrane phospholipids and overall membrane integrity and cell growth [32]. In this context, cell growth refers to an increase in the cell surface area-to-volume ratio, a factor with significant implications for membrane capacitive properties. The relationship linking capacitance to cell membrane area was previously demonstrated in experimental research employing a biophysical membrane model approach, specifically focusing on artificial lipid bilayers composed of egg phosphatidylcholine, cholesterol, and n-decane [33].

All membrane-bound organelles (e.g., endoplasmic reticulum, Golgi apparatus, mitochondria, nuclear envelope) display similarities with the cell membrane regarding their lipidic composition and organization as BLM, potentially functioning as dielectric materials capable of storing electrical charge [27]. However, further evidence is needed to prove that these cellular components can act as true capacitors. If proven, higher levels of capacitance might be found in the membranes of organelles that have a relatively pure lipid composition, which is often observed in the early stages of life [34]. However, as age progresses, genetic mutations and environmental stressors lead to the aggregation of abnormal proteins within the internal cellular membranes, disrupting membrane integrity and signaling pathways [35] and potentially diminishing its capacitive properties.

##### Proteins

In the human body, the key location of proteins includes the cell membrane, cytoplasm, nucleus, and organelles, where a diverse range of structural and signaling processes occur. Despite these molecules not inherently exhibiting capacitive characteristics in the same way as others (e.g., lipids), their constitution and functional roles contribute to characterize them as facilitators of intracellular conductivity. From a biophysical perspective, great attention has been given to proteins coupled in the cell membrane – i.e., voltage-gated ion channels. When facing voltage, mechanic, or binding-related stimulus, these particular proteins act as channels that selectively or non-selectively allow inorganic ions with electrical charge to cross the membrane, thereby representing the primary mechanism to enhance conductance across the membrane [20]. This process follows the principle of *ion selectivity* and can be found in various protein complexes responsive to external electric current – i.e., Na^+^, K^+,^ and Ca^2+^ ion channels [20]. When an external electrical stimulation, operating within the optimal frequency range (e.g., kHz), reaches the outside of the cell membrane, two sequential events favoring cell membrane depolarization occur. While there is an initial low current flow through leak channels in a passive manner, as the electrical stimulus builds up across the cell membrane, distinct ion-selective channels actively open, allowing positively charged ions to migrate according to the electrochemical gradient.

Although this physiological process is not expected to impact intracellular electrical conductivity, as it only represents the primary mechanism to allow current flow within the cell, previous evidence has shown that it slightly enhances membrane capacitance. This is relevant when using frequencies close to the low-frequency end of the surface membrane (i.e., 500 Hz). This was demonstrated in animal experiments, where the voltage-dependent capacitance of isolated muscle fiber membranes from *post-mortem Rana pipiens* was shown to increase by approximately 5% in response to 500 Hz frequency without the prepulse [36]. According to the author, it is likely that the origin of charge displacements in muscle membranes relies on the multiple ion channels (e.g., Na^+^ and K^+^) and non-linear processes (e.g., Ca^2+^ and Cl^−^ fluxes) [36]. When translating these findings to humans, given the high abundance of ion-channel proteins, efficient cellular signaling processes, and limited exposure to oxidative stress in youth [37], it is plausible that mechanisms enhancing capacitance in response to similar frequencies (e.g., multifrequency devices) are refined in early ages. However, an accompanying exposure to reactive oxygen species with aging can cause protein misfolding and aggregation [37, 38], further impacting overall cells’ electrophysiology. This also applies to other cytoskeleton proteins, including tubulin, which have also been highlighted as important electrical resistors [39]. Recent in vitro experiments have highlighted that elevated concentrations of tubulin, i.e., polymerized microtubules accompanied by increased counter-ion concentrations, directly contribute to enhancing intracellular conductivity [40].

##### Others

Despite the evidence highlighting lipids and proteins as the most relevant organic elements of electrical capacitance and conductivity, the biophysical implications of other organic molecules deserve consideration. Already in 1906, Zuntz et al. [41] demonstrated that glycogen, the most common molecular form of carbohydrates in humans, exhibits water-binding properties by changing the surrounding ionic environment. Considering the strong affinity of glycogen with high molecular weight for water molecules at a proportion of 1:3 g (i.e., water binding) [42, 43], one can expect glycogen to have a positive relationship with electrical conductivity. Particularly in children and adolescents, who typically require higher demands for energy storage in the form of intracellular glycogen to support growth and metabolic processes [44], it is reasonable to anticipate that increased glycogen levels will yield a rise in ICW. This physiological adaptation alone can, in turn, enhance ions’ mobility within the cell, favoring intracellular electrical conductivity [45]. On the other hand, the amplified presence of intracellular glycogen in younger individuals also contributes to arranging water molecules as “hydration shells” (i.e., water molecules with the negatively charged oxygen atoms binding to the carbohydrate molecule and the positively charged hydrogen atoms facing away). From a biophysical perspective, this intracellular compound reduces the ability of the cell environment to insulate or polarize [35], making the intracellular medium less effective in resisting the flow of electric current.

Similar to glycogen, the presence of creatine, as an osmotically active substance, is expected to increase ICW retention in muscle cells by drawing water into the intracellular space [46]. Although this effect is particularly evident after creatine loading, resulting in temporarily decreased intracellular conductivity and increased muscle volume across all ages, it is not clear whether creatine supplementation alters TBW relative to muscle mass over the long term [47].

#### Water

Despite ICW representing the central water compartment in the body (i.e., ~ 60% of TBW), great attention has been placed on quantifying and qualifying ECW, which compromises approximately 92% of all extracellular fluids (i.e., cellular-level) [48], thus representing around 40% of TBW [49]. Often regarded as the combined volume of plasma and interstitial fluids - i.e., two fluids with high conductive properties owing to their abundant electrolyte composition [50], ECW has been highlighted as the primary determinant of conductivity in the human body. Contrary to ICW, whose intracellular pathway primarily depends on the capacitive effects of BLM, the ECW pathway is known to be purely resistive. As a result, the resistivity of ECW is expected to differ from the ICW by a factor of 3 to 4, regardless of the frequency range employed [51]. Notwithstanding that increased levels of ECW per se may contribute to increase conductivity, evidence suggests that the rise in ECW proportion relative to ICW (ECW: ICW) may also reflect a state of decreased cellular health (i.e., loss of BLM capacitive properties) [52]. In line with this, 50 kHz BIA-derived PhA has been found to be inversely associated with several protein markers such as adrenomedullin and N-terminal prohormone brain natriuretic peptide, which are implicated in the proteomic profile of heart failure – i.e., a condition often characterized by overhydration and altered fluid distribution [53]. On the other hand, the decreased absolute ECW levels may suggest systemic dehydration and fluid loss [52], which impacts PhA mostly due to loss of conductivity (i.e., dominating mechanism). This is particularly true in conditions where ECW decreases independently of changes in ICW (e.g., acute dehydration; suggesting maintenance of capacitance properties). Interestingly, this event can also reflect improved cellular health (e.g., improved cellular metabolism and protein synthesis), as well as better hydration balance (e.g., water retention through glycogen and creatine). Particularly in this last scenario, the shift towards a higher proportion of ICW relative to ECW (i.e., cell swelling phenomena) is expected to be positively associated with PhA, as this association is primarily driven by the capacitive properties resulting from the integrity of cell membranes [54].

While ECW accounts for most of TBW in fetuses, the ratio between ECW and ICW progressively falls during childhood to the point where it stabilizes until young adulthood (i.e., a ratio of 3:2) and ICW becomes the significant component of TBW (i.e., ~ 60% of TBW) [55, 56]. Due to the physiological changes between sexes, favoring SMM and BCM accumulation in males [19, 57], the ECW content decreases from around 43–40% in males from 3 to 18y of age [58]. Conversely, females maintain their proportion of ECW (i.e., ~ 42%) relatively stable during the same development stages [58]. Until puberty, when the absolute volume and relative portion of ECW in females match the numbers of males, similar levels of electrical conductivity within the body would be expected. However, recent evidence using a tetrapolar 50 kHz frequency device demonstrated that males exhibit higher levels of conductivity than females [59], eventually opening the discussion to potential sex-specific qualitative factors impacting conductivity. As age progresses, males, in particular, shift towards greater ICW, slightly contributing to decreased ECW: ICW ratio [58]. Despite the decrease in ECW: ICW ratio initially suggesting a decrease in conductivity, the available evidence clearly shows that it is not merely the ratio but the absolute of ECW that strongly influences conductivity [60]. Furthermore, this rise in ICW is expected to reflect an increasing number of cells and, consequently, a larger amount of cell membranes. This expansion enhances capacitance, further contributing to the observed PhA increases with age.

### Cellular-level determinants

While moving up the scale of biological complexity, attention must be given to major biological components sustaining cellular levels with implications on how the electric current flows through the body. Amongst the various interrelated factors influencing raw BIA parameters, particular attention will be given to the biophysical significance of BCM (Fig. 2, **Cellular**), which includes the full spectrum of cells exclusively containing intracellular fluids and solids [61, 62]. BCM has been proposed as all the cells of the body capable of oxidizing substrates to obtain or convert energy - i.e., oxygen-requiring, carbon-dioxide-producing, glucose-burning mass of tissue - and reproducing through mitosis processes [61]. Despite being true that BCM will be predominantly influenced by the cell content of larger organs, such as the muscle tissue (i.e., myocytes account for up to 80% of BCM), the characteristics of other cells (e.g., adipocytes) may also play a role on the relationship of BCM and raw BIA variables [62, 63].

Due to the inherent characteristics of BCM in the human body (i.e., 20–55% of body mass), which compromises approximately 85% of ICW and 15% of cell solid content [62], two direct implications of this component can be seen in the electric current flow. First, due to the high proportion of ICW content, previous evidence has consistently shown that increased BCM can enhance overall electrical conductivity [23]. When looking at additional underpinnings of BCM, the available evidence stands out that increased portions of cell solids (e.g., lipid and protein structures localized at the BLM and within the cell) may also improve bioelectrical properties of the body by favoring overall cell capacitance and electrical conductivity [36, 39], potentially suggesting an increase in PhA. While the positive relationship between BCM and PhA has been well established over time [60], recent findings offer a novel perspective, demonstrating that protein markers, including myoglobin, matrix metalloproteinase-9, and protein-glutamine gamma-glutamyltransferase, which are strongly linked to muscle mass and, consequently, BCM, also exhibit a positive association with PhA (50 kHz frequency BIA device) [53]. Expanding on this relationship, other investigations using a tetrapolar 50 kHz frequency BIA device highlighted that BCM may have a more pronounced impact on other raw BIA variables, such as Xc in parallel (i.e., an indicator of ICW) and capacitance (i.e., an indicator of cell mass) [64]. The authors demonstrated that both variables are expected to display strong relationships with ICW [64] and, hence, BCM, potentially even more so than PhA. These findings underscore the need for further research to identify raw BIA variables that should be targeted when predicting specific components of the body.

#### Skeletal muscle cells – myocytes

Great interest has been placed on skeletal muscle fibers, not only for representing the highest portion of muscle cells [63] - i.e., at the age of 17y, more than two-thirds of BCM is composed of muscle fibers (i.e., 69%) [57, 65], but also because of their high plasticity and metabolic activity. With these factors potentially interfering with the dielectric properties of the human body, several investigations examined the biophysical impact of specific characteristics of these cells (e.g., muscle fiber length, girth, and type). Previous evidence has demonstrated that muscles have most of their fibers arranged in parallel arrays along the muscle’s longitudinal axis, potentially representing a source of enhanced conductivity compared with other muscle type arrangements. While longer muscle fibers usually observed in parallel muscles may provide more surface area for current flow with influence on the capacitive properties of this tissue, the increased length typically results in lower conductivity [66]. This knowledge follows the principle that in a uniform conductor with constant geometry and biological composition (e.g., resistivity and CSA), the conductivity is directly associated with the length of the segment [1, 67, 68]. When looking at other muscle shapes with shorter muscle fibers, such as the pennate muscles, an opposite trend can be expected in markers of conductivity. This fact, together with the anisotropic characteristics of distinct muscle shapes previously described, suggests a “conductivity paradox”. On the one hand, muscles with a parallel arrangement of fibers are more aligned but have generally less and longer muscle fibers compared to pennate muscles, and on the other hand, pennate muscles have an oblique arrangement of fibers but show a more significant amount of muscle fibers, in particular muscle fibers of reduced length.

To minimize these confounding effects of muscle fiber size and shape on whole-body BIA assessments, several authors have proposed using multiple voltage-sensing electrodes positioned along the muscle, allowing for the extraction of spatial dependencies in localized BIA [69, 70]. This geometric approach facilitates the development of mathematical models that connect impedance data to underlying muscle anatomy, improving the accuracy of muscle composition assessments. In this context, there has been particular interest in current flow transverse to muscle fibers, as it encounters more cell membranes per unit distance [70], making it a potentially more informative diagnostic parameter of cells, especially in health-compromised populations (e.g., muscle atrophy and injury).

Also, depending on the muscle type and other external factors (e.g., sports participation and injuries), the CSA of muscle fibers can change significantly, potentially affecting regional and whole-body measures of conductivity and capacitance [71]. Because muscle fibers have distinct morphology - i.e., type I fibers are mainly oxidative with quantities of large mitochondria and water, while type II fibers mainly rely upon glycolytic processes with considerably lower mitochondria [72], and volume – e.g., in adulthood, the CSA of type I and II fibers corresponds, respectively, to 4726.7 µm^2^ and 5395.5 µm^2^ [73], some differences can be expected in biophysical properties of capacitance and conductance. Although no evidence exploring this relationship exists in humans, data from *post-mortem* animal investigations showed that muscle complexes with predominantly type I fibers exhibited increased conductivity than type II fibers, mainly when conducting experiments with low-frequency data and with muscle fibers disposed longitudinally [72]. These findings support a more significant influence of water proportion (type I fibers) than the volume of type II fibers; however, this effect is lost when conductivity is analyzed in muscle fibers displayed in transverse. Still, in *post-mortem* animal models, other studies have shown that capacitance is greater in fast-twitch fibers than in slow-twitch fibers [74], likely due to their larger volume and, consequently, a higher cell surface area-to-volume ratio, which involves naturally more lipids and protein structures (i.e., biological capacitors).

When looking at muscle cell characteristics in youth, one can observe that children and adolescents exhibit significantly lower muscle fiber lengths than adults, with the magnitude of these differences varying by muscle region, particularly in females [75]. Despite this fact suggesting a potential reduction in the electrical current path and, consequently, in conductivity, it may negatively impact the capacitive properties of this tissue, potentially influencing PhA. Nevertheless, the muscle fiber length does not remain constant through childhood, as it increases significantly until puberty but only slightly after that, perhaps suggesting that overall muscle mass increases due to changes in the CSA of fibers during adolescence [76]. Although both types of fibers exhibit similar CSA in youth [77, 78], the proportion of type I fibers remains significantly higher than that of type II fibers [77, 79], favoring overall conductivity within the body. While there is still a significant amount of type II fibers in youth, which could emerge as an index of reduced conductivity, these fibers appear not to be fully matured (i.e., less myofibrils and less water availability), potentially affecting the dielectric properties of BCM. Further evidence is needed to understand how intracellular age-related changes in fiber types occur and how they impact the conductivity and capacitive properties of tissue. At this level, the localized BIA emerges as a novel approach, offering limb-specific measurements of raw parameters with the potential to detect changes in fiber composition [70].

#### Adipose cells – adipocytes

Various forms of adipocytes contributing prominently in size to overall cell mass were shown to affect the electrically conductive path in different magnitudes. Despite the lack of evidence exploring the dielectric properties of different types of adipocytes in humans, data from an investigation conducted in *post-mortem* animals showed that brown adipose cells exhibited nearly twice the capacitive properties compared to white adipose cells [80]. Despite brown adipose cells having a smaller size than white adipose cells [81], their higher organelle density, particularly the abundance of mitochondria with capacitive structures (e.g., lipids and proteins), may contribute to increased electrical current storage [82, 83]. Although it was expected that conductivity would show similar patterns, considering the greater volume of water within brown adipose cells [82], no significant differences were reported between adipocytes, particularly at lower frequency ranges (e.g., kHz) [80]. A possible explanation for this finding is that the proportion of lipids remains relatively high in both types of adipocytes (i.e., a single cytoplasmic lipid droplet in white adipocytes and multiple cytoplasmic lipid droplets in brown adipocytes) acts as an insulating factor, limiting current flow throughout the cell.

Age-related changes in adipocytes occur mainly due to internal variations within these cells, potentially affecting cells’ biophysical characteristics. Data from both animal [84] and human [85] models studies have demonstrated a direct link between adipocyte size, volume, and age, with these findings being likely attributed to increases in specific intracellular components (i.e., lipid droplets) over time. Due to higher hormonal secretion (e.g., adiponectin) and energy allocation toward critical processes like growth [83], children and adolescents are expected to have lipid droplets of smaller sizes. This knowledge helps us understand the previous findings that smaller mature adipocytes are observed in young ages [84, 85]. Moreover, it prompts a new discussion on whether conductive and capacitive characteristics may be positively influenced as a result of less intracellular fat accumulation in youth, along with a compensatory increase of other organelles with capacitive properties.

### Tissue-level determinants

Moving forward from cellular determinants of raw BIA variables to others at the tissue level, particular attention is given to SMM. Although the human body is composed of other tissues, including adipose tissue, bone mass, blood, and organ-specific tissues, representing approximately half of the body mass in adulthood, we further describe qualitative factors related to SMM at the tissue level relating to dielectric properties (Fig. 2, **Tissue**).

*Tissue orientation*. In highly conductive tissues, such as SMM, the arrangement of biological structures (e.g., muscle fiber orientation) plays a key role in determining their conductive properties. Following the initial experiments during the 1870s, which first unveiled the tropic characteristics of muscle tissue [86], several investigations focused on describing the impact of these properties on electrical conductivity. In the animal model, for example, the conductivity of the muscle measured in a direction parallel to the fiber orientation is ten times higher than that measured perpendicular to the fiber axis (i.e., ~ 7 mS.cm ^− 1^, parallel direction; <1 mS.cm ^− 1^, perpendicular direction; Hz) [87]. While this phenomenon holds at audio frequencies (i.e., kHz), where muscle conductive properties exhibit strong anisotropy [68, 87], as the frequency range approaches MHz, the conductivities measured in both directions converge to identical values (i.e., ~8mS.cm ^− 1^). Recent investigations conducted in similar conditions in humans using localized BIA at the lower limb corroborate these findings, suggesting a decrease in muscle anisotropy beyond 100 kHz [88].

Under the assumption that muscle complexes aligned with the electric current path present lower anisotropic characteristics, some authors have explored this by comparing the resistivity levels of muscles with different orientations using localized tomography. In brief, muscles with oblique or transversal orientations relative to the direction of the current flow path have approximately 15–20% lower conductivity than muscle complexes mostly arranged in parallel [89]. Due to the body’s anisotropic properties, this unique orientation may negatively affect local conductivity and undermine PhA. Despite the electrical conductivity also being expected to be lower in other major muscles not fully aligned with the electric current path, further research is needed to clarify how muscles’ orientation may interfere with BIA assessments.

*Tissue Architecture.* Because the human body exhibits a unique architectural anatomy with multiple muscle fiber arrangements, which can directly impact electrical conductivity within the body, an extensive effort has been made to classify muscle shapes into distinct categories – i.e., parallel, pennate, convergent, and circular. Due to the anisotropic characteristics of muscle tissue, when exposing the body to electrical stimulation in the range of Hz and kHz, the conductivity is expected to be enhanced in the parallel muscles but not in muscles with other fiber arrangements (e.g., oblique and convergent). Although previous investigations using localized electromyography in the upper [90] and lower limb [91] demonstrated that the amplitude of the signal traveling decreases as the pennation angle of muscle fibers increases, no investigations to date have explored the direct impact of different muscle shapes or fiber arrangements on BIA electrical conductivity under resting conditions.

Despite muscles’ orientations remaining largely stable throughout the lifespan, assuming a uniform interpretation of electrical conductivity and raw BIA variables within this biological tissue may not be appropriate, particularly when comparing populations with specific muscle architectures, such as children and adolescents. Even though dielectric properties of the muscle tissue derive mostly from its composition of myocytes at the cellular level, which relies largely on atomic-molecular components with capacitive (e.g., lipidic structures of the BLM) and conductive properties (e.g., inorganic ions; ICW), specific muscular arrangements in youth can have an impact in these properties. For example, Binzoni and colleagues [92] demonstrated that the human pennation angle is not constant during growth but increases as a function of age until the first decade of life. Using localized ultrasound analysis, the authors showed that the average *gastrocnemius medialis* pennation angle increases around 5 degrees from the age of 5y to 10y, at which point it stabilizes [92]. Additional studies showed no trend of increase in the pennation angle of other muscle complexes after the age of 10 [65, 66]. Taking these findings into account, together with electrical conductivity determinants, the rise in the pennation angle through childhood may increase anisotropy properties, thus contributing to a gradual decrease in electrical conductivity. This fact contradicts the tendency towards increasing conductivity throughout growth [93], suggesting that there are more relevant factors in determining electrical conductivity in muscle tissue. However, while whole-body BIA assumes uniform electrical properties and overlooks changes in muscle architecture due to growth, localized BIA presents a promising alternative by capturing site-specific variations in muscle orientation and structure, which may be particularly relevant for younger children. Nevertheless, further research is needed to confirm its accuracy in assessing growth-related changes and muscle anisotropy in the pediatric population.

## Multilevel body composition determinants of phase angle in youth – quantitative perspective

**Fig. 3:**
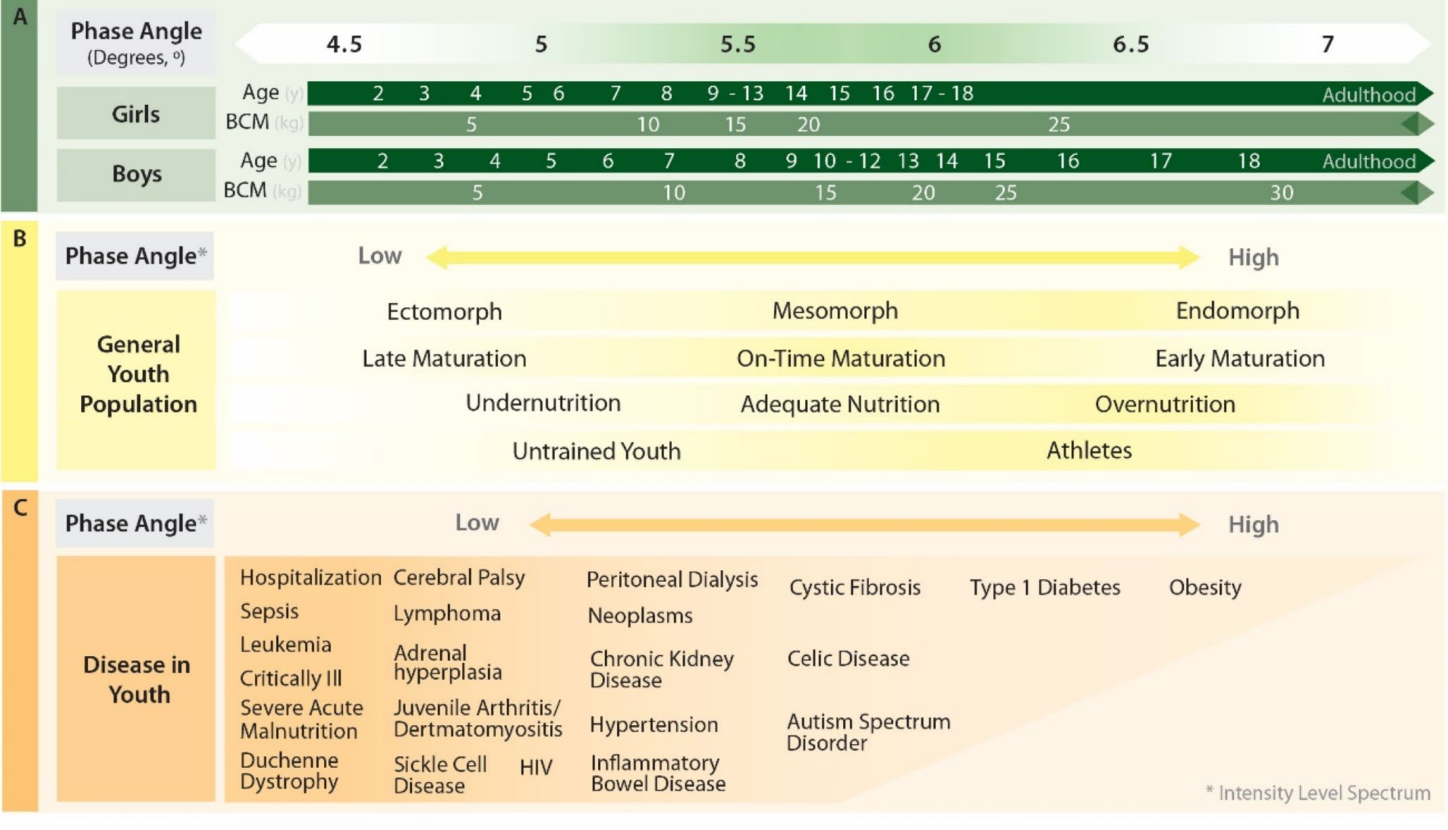
Proposed characterization of phase angle in youth: (**A**) shows its relationship with biological features of age and body cell mass; (**B**) illustrates its relationship with physical morphology, maturation and nutrition status, and training habits; (**C**) depicts its relationship with multiple diseases

While describing qualitative determinants across body composition levels helped clarify the biological influence on electrical conductivity and capacitance, further research is needed to understand how these determinants manifest on a larger scale in response to quantitative changes during youth. As childhood and adolescence are marked by significant cellular and tissue growth – through hyperplasic and hypertrophic processes – the demand for essential cellular components also rises, resulting in elevated amounts of inorganic ions and organic and water molecules (Fig. 3, **panel A**).

Because the highest rates of increase in capacitive and conductive properties in the human body are observed in the early life period, significant attention has been given to PhA for combining multiple biophysical properties. In youth, several reference values regarding PhA measurements obtained from tetrapolar 50 kHz frequency BIA devices have been recently proposed [16, 19]. According to Mattiello et al. [16], PhA rapidly increases by approximately 2º from infancy until 5 years of age in both sexes (i.e., males, 3.6º to 5.6º; females, 3.1º to 5.4º). Immediately after, the increasing rate of PhA slows down until puberty, when males and females exhibit values ranging from 6.1º to 6.6º and 5.7º to 6.1º, respectively. Although both sexes continue to increase PhA after puberty, males experience a more pronounced rise, with an average increase of 0.9º until 18 years (i.e., 7.0º to 7.5º) [16]. In contrast, females exhibit an increase of only 0.5º until adulthood – i.e., when PhA typically lies between 6.1º and 6.8º. To provide a deeper understanding of quantitative changes in PhA, we provide an explanatory framework exploring the link and logical dependence of this raw BIA parameter on relevant biological structures, such as BCM, as well as other tissues with distinct conductive and capacitive characteristics (i.e., skeletal muscle and adipose tissue).

Despite BCM remaining relatively stable between the 20s and 30s - i.e., the moment at which the PhA is maximized [16] – significant changes can be observed in most cellular components during youth [14] (Fig. 3, **panel A**). From early childhood (i.e., 4-5y) to young adulthood, absolute BCM levels increase around 3 to 4-fold in both sexes (i.e., males, ~ 8 to 32 kg; females, ~ 7 to 24 kg), favoring an overall increase in PhA assessed with a tetrapolar 50 kHz frequency BIA device (i.e., males, ↑2º; females, ↑1º) [12]. With early investigations showing that overall SMM represents up to 80% of the total BCM [62], it is apparent that both components are expected to progress at similar rates until young adulthood (i.e., 5–7 y to + 17y; males, ~ 6 to 25 kg; females, ~ 6 to 18 kg) [94, 95], thus affecting biophysical properties comparably. From the multiple factors contributing to BCM expansion in youth, beyond those related to hyperplasia-driven growth, attention must be given to hypertrophic processes, such as the increase in the CSA of muscle fibers and a consequent rise in absolute levels of ICW, with the potential to enhance both conductive and capacitive properties. Notwithstanding the increase in muscle fibers’ CSA by 2 to 3-fold from birth to young adult ages [57], distinct rates of increase must be expected regarding the type of muscle fiber and development period [78].

Although males and females exhibit similar levels and rates of increase of BCM until puberty, which helps to explain the similar levels also observed in PhA (i.e., 4-5y: 5.0º in males and females; 6-10y: 5.5º in males, 5.3º in females; tetrapolar 50 kHz frequency BIA device), significant differences between sexes, mainly attributed to the physiological growth processes (e.g., hormonal changes), are observed thereafter [12, 15]. While BCM continues to increase at a similar rate in both sexes during the growth spurt stage when significant physical changes are expected, PhA remains relatively constant (i.e., males, 5.7º; females, 5.3º) [12]. Among the factors explaining this situation, the most likely may be that the increase of BCM, which enhances capacitive properties within the body, may be obscured by a potential interference with the lengthening of the electric current path (i.e., lower conductivity) resulting from a spontaneous increase in stature and body segments.

While BCM continues to increase until the onset of adulthood, at which point it begins to stabilize, it is at this moment – i.e., adolescence – that significant changes in PhA occur. The delay in PhA increase may be not only explained by the increase in the relative contribution of overall SMM after the growth spurt (i.e., three times higher in males than females) [95]. With the maximum rate of increase in type I fibers and major differentiation processes of type II fibers occurring immediately after puberty [78], a progressive improvement in PhA can be expected (e.g., males, 11% increase). It is also at this stage that changes in other biological parameters take place. For example, following the adipose tissue cellular proliferation during the growth spurt [96], significant differences can be observed in adipose tissue in both sexes. While males exhibit a 2-fold increase between 11 and 18y (i.e., 4.7 to 9.2 kg), a 3-fold increase in adipose tissue can be observed in the female population within the same period (i.e., 6.0 to 17.1 kg) [58]. As a result of an increase in adipose cells and qualitative changes (e.g., increase in intracellular fat content) – i.e., important modulators of electrical conductivity and capacitance [15] - the biophysical properties, in particular R, may be negatively impacted. Particularly in females, these biological traits contribute to the lowest rates of age-related PhA increase observed after puberty (i.e., only a 1.5% increase) [12, 19]. Overall, both phenomenons explain the 8% higher PhA values observed in males than females after puberty (i.e., males, 6.1–6.6º; females, 5.7–6.1º; tetrapolar 50 kHz frequency BIA device) [16, 19, 97].

Beyond the innate developmental progression in BCM and other biological components highlighted in Table 1, it is noteworthy that several intrinsic (e.g., body phenotype and maturation status) and extrinsic factors (e.g., nutritional intake and physical activity) may impact the body composition of youth [60]. Because these specific attributes have potential implications for overall levels of BCM and other tissues, most raw BIA variables are expected to vary, even among young individuals of similar ages (Fig. 3, **panel B**). Furthermore, it is important to highlight that in specific populations, such as athletic and health-compromised youth, PhA is influenced by multiple physiological changes. While trends in athletes have already been extensively explored [98], it is essential to better understand how PhA behaves in youth with different health conditions to refine its clinical application and interpretation.

**Table 1:** Plausible qualitative and quantitative determinants of dielectric properties and their potential impact on phase angle in youth

Qualitative Determinants†		Implications on dielectric properties	Expected influence on PhA
**Atomic – Molecular level**			
Inorganic ions	Unclear*	-	-
Organic molecules: Lipids	Higher proportion of fatty acids	↑ Capacitivity	↑ PhA
	Increased cell surface area-to-volume ratio, resulting in a more significant proportion of BLM per unit cell volume	↑ Capacitivity	↑ PhA
	Organelles’ membranes have a relatively pure lipidic composition	-	-
Organic molecules: Proteins	Higher proportion of ion-channel proteins	↑ Capacitivity	↑ PhA
	Upregulated expression and synthesis mechanisms of tubulin	↑ Conductivity	↑ PhA
Organic molecules: Carbohydrates	Higher demands for energy storage to support growth	↑ Conductivity	↑ PhA
	Higher proportion of intracellular glycogen in the form of hydration shells	↑ Capacitivity	↑ PhA
Water	Higher ECW: ICW ratio	↑ Conductivity	↑ PhA
**Cellular level**			
Muscle cells	Shorter muscle fibers length	↑ Conductivity ↓ Capacitivity	↓/↑ PhA
	Shorter muscle fibers CSA	↓ Conductivity ↓ Capacitivity	↓ PhA
	Higher proportion of type I fibers	↑ Conductivity	↑ PhA
	Immaturity of type II fibers	↓ Conductivity	↓ PhA
Adipose cells type	Higher proportion of brown adipose cells possessing more capacitive structures	↑ Capacitivity	↑ PhA
	White adipose cells with smaller lipid droplets	↑ Conductivity	↑ PhA
**Tissue level**			
Skeletal muscle tissue: Orientation	Unclear*	-	-
Skeletal muscle tissue: Architecture	Lower pennation angles	↑ Conductivity	↑ PhA
**Quantitative Determinants**†			
**Atomic – molecular level**			
Inorganic ions	Lower levels of inorganic ions (e.g., Na^+^, K^+^, Mg^2+,^ Ca^2+^, HPO_4_^2−^)	↓ Conductivity	↓ PhA
Organic molecules: Lipids	Lower lipid levels and organelles availability	↓ Capacitivity	↓ PhA
Organic molecules: Proteins	Lower protein levels	↓ Capacitivity	↓ PhA
Organic molecules: Carbohydrates	Lower carbohydrate levels	↓ Conductivity	↓ PhA
Water	Lower levels of TBW, ECW, and ICW	↓ Conductivity	↓ PhA
**Cellular level**			
Body cell mass	Lower levels of BCM	↓ Capacitivity ↓ Conductivity	↓ PhA
Muscle cells	Lower levels of all muscle cell types	↓ Conductivity	↓ PhA
Adipose cells	Lower levels of adipose cells	↑ Conductivity	↑ PhA
**Tissue level**			
Skeletal muscle tissue	Lower quantity of muscle tissue	↓ Capacitivity ↓ Conductivity	↓ PhA
Adipose tissue	Lower quantity of adipose tissue	↑ Conductivity	↑ PhA

Abbreviations: BCM, body cell mass; BIA, bioelectrical impedance analysis; BLM, bilayer lipid membrane; CSA, cross-sectional area; ECW, extracellular water; ECW:ICW, extracellular to intracellular water ratio; ICW, intracellular water; PhA, phase angle; TBW, total body water

† Qualitative and quantitative determinants of children and adolescents compared to adults (i.e., + 18 years old)

* Lack of evidence highlighting the impact of multilevel determinants on raw bioelectrical impedance variables

## Multilevel determinants of raw BIA variables in health-compromised youth

Important considerations must be highlighted when tracking PhA and other BIA variables in health-compromised youth populations. Despite being commonly accepted that a lower PhA is a direct marker of unfavorable health conditions, this association is not applicable to all diseases, as shown in Table 2. Most evidence suggests lower PhA in hospitalized youth or pediatric patients compared to healthy controls [99–114], however, variations exist across specific diseases. For instance, youth diagnosed with type I diabetes [115, 116], obesity-related [117–121], and stunting conditions [122] are expected to have higher PhA values compared to healthy age-matched controls, while pediatric patients with bowel disease, celiac disease, or cystic fibrosis tend to show similar PhA values to healthy individuals [123–128]. Of the various reasons that could explain this situation, the most logical seems to be that, despite a possible negative influence of a particular disease on some biophysical parameters, there seems to be a compensatory effect on other properties that mitigates this impact (Fig. 3, **panel C**). Taking obesity-related diseases as an example, although an increased number of adipose cells and tissue can compromise electrical conductivity within the body, a higher amount of BCM may compensate by enhancing capacitance to a level that even favors PhA. Nevertheless, absolute values in Table 2 should be interpreted with caution, as direct comparisons across investigations may be influenced by technical differences between BIA devices. Therefore, the comparisons provided should be considered indicative trends rather than absolute benchmarks, ensuring that methodological nuances are carefully accounted for in both research and clinical applications.

Since most evidence directly focuses on distinct etiological factors causing quantitative and qualitative changes in biological structures and disruptive mechanisms in water distributions in the context of congestion or inflammation to explain impaired raw BIA variables in response to unhealthy conditions [8], there is still limited understanding on the impact of changes in biological elements of lower levels (Fig. 2), which often cascade upward, influencing higher-level components. Due to the implications of each disease on multiple level components, potentially affecting dielectric properties and, consequently, PhA in different ways, we recommend that authors carefully manage the interpretation of PhA and emphasize the importance of looking at the shifting trends in other relevant BIA variables in response to disease-specific conditions in youth.

**Table 2:** Comparison of phase angle and raw bioelectrical impedance analysis-derived variables across multiple diseases in youth and healthy controls

Autoimmune and immunodeficiency diseases							
*Authors*	*Country*	*Population*	*Investigation groups*	*Sample*,* n females*	*Device*	*BIA*	*Outcomes (Mean ± Standard Deviation; IQR)*
Wiech, 2018 [98]	Poland	Juvenile idiopathic arthritis	**Con**: 12.8 ± 3.9y (F), 12.4 ± 3.7y (M) **G1**: 12.9 ± 4.0y (F), 12.4 ± 3.7y (M)	**Con**: 46, 34 F **G1**: 46, F	BIA 101, Akern; SF, 50 kHz; 4-electrode	R	R, **Con**: 656.2 ± 94.8, **G1**: 699.6 ± 118.0, **Con**: 679.6 ± 74.5, **G1**: 714.3 ± 104.3 (F), **Con**: 589.1 ± 116.1, **G1**: 657.9 ± 147.5 (M);
						Xc	Xc, **Con**: 66.3 ± 7.5, **G1**: 66.3 ± 12.2, **Con**: 67.4 ± 7.7, **G1**: 65.9 ± 9.3 (F), **Con**: 63.3 ± 6.2, **G1**: 67.6 ± 18.7 (M)
						**PhA**	PhA, **Con**: 5.9 ± 0.8, **G1**: 5.5 ± 0.6*, **Con**: 5.7 ± 0.7, **G1**: 5.3 ± 0.5 (F)*, **Con**: 6.3 ± 0.9, **G1**: 5.9 ± 0.8 (M)
Pugliese, 2023 [99]	Brazil	Juvenile dermatomyositis	**Con**: 10.7 ± 3.1y **G1**: 11.8 ± 4.0y	**Con**: 24, 14 F **G1**: 30, 18 F	450, Biodynamics; SF, 50 kHz; 4-electrode	**PhA**	PhA, **Con**: 6.1 ± 1.0, **G1**: 5.2 ± 1.3*
**Cardiovascular diseases**							
Švigelj, 2021 [117]	Slovenia	Hypertension	**Con**: 13y with 7y IQR^†^ **G1**: Healthy weight hypertension, 16y with 4y IQR^†^ **G2**: Obesity-related hypertension, 14.5y with 4y IQR^†^ **G3**: Chronic kidney disease^‡^, 10.0y with 8y IQR^†^	**All**: 128, 57 F **Con**: 38, N/A F **G1**: 30, N/A F **G2**: 30, N/A F **G3**: 30 N/A F	Nutrilab, Akern; SF, 50 kHz; 4-electrode	**PhA**	PhA, **Con**: 5.8 with 1.2 IQR^†^, **G1**: 6.7 with 1.4 IQR^†^*, **G2**: 6.5 with 1.2 IQR^†^*, **G3**: 5.8 with 1.0 IQR^†^
**Gastrointestinal diseases**							
Werkstetter, 2012 [124]	Germany	Crohn’s disease and ulcerative colitis	**Con**: 15.2 ± 3.1y **G1**: 15.1 ± 2.9y	**Con**: 39, N/A **G1**: 39, 25 F	Nutriguard M, Data Input; MF; 4-electrode	**z-PhA**	PhA, **Con**: 0.09, **G1**: -0.64
Wiech, 2018 [100]	Poland	Crohn’s disease and ulcerative colitis	**Con1**: 13.5 ± 3.4y **G1**: Ulcerative Colitis, 13.5 ± 3.4y **Con2**: 13.8 ± 3.1y **G2**: Crohn’s disease, 13.8 ± 3.1y	**Con1**: 34, 17 F **G1**: 34, 17 F **Con2**: 25, 8 F **G2**: 25, 8 F	BIA 101, Akern; SF, 50 kHz; 4-electrode	R	R, **Con1**: 623.7 ± 106.3, **G1**: 654.2 ± 130.4, **Con2**: 606.6 ± 103.7, **G2**: 700.7 ± 118.5*;
						Xc	Xc, **Con**1 64.2 ± 8.2, **G1**: 61.7 ± 13.1, **Con2**: 61.9 ± 6.9 **G2**: 63.4 ± 10.4;
						**PhA**	PhA, **Con**1: 6.0 ± 0.8, **G1**: 5.3 ± 1.3*, **Con2**: 5.9 ± 0.6, **G2**: 5.2 ± 1.2*
Wiech, 2018 [122]	Poland	Celiac disease	**Con**: 10.6 ± 4.0y **G1**: 10.8 ± 4.0y	**Con**: 41, 20 F **G1**: 41, 20 F	BIA 101, Akern; SF, 50 kHz; 4-electrode	**PhA**	PhA, **Con**: 5.6 ± 0.8, **G1**: 5.5 ± 0.7
**Genetic diseases**							
Pileggi, 2016 [101]	Brazil	Osteogenesis imperfecta	**Con**: 116.1 ± 39.8 m **G1**: 104.7 ± 52.4 m	**Con**: 17, 9 F **G1**: 7, 5 F	N/A	R	R, **Con**: 728.6 ± 110.4, **G1**: 690.6 ± 158.3;
						Xc	Xc: **Con**: 69.5 ± 0.6, **G1**: 56.7 ± 15.4*;
						**PhA**	PhA, **Con**: 5.5 ± 0.6, **G1**: 4.7 ± 0.9*
**Hematological diseases**							
Farias, 2012 [125]	Brazil	Hematological, onco-hematological diseases	**Con**: N/A **G1**: 10.2 ± 4.1y	**Con**: 38, N/A F **G1**: 67, 28 F	Quantum II, RJL; SF, 50 kHz; 4-electrode	**sPhA**	sPhA, **Con**: 1.0 ± 0.6, **G1**: 0.6 ± 1.0
VanderJagt, 2002 [102]	Nigeria	Sickle cell disease	**Con**: 12.9 ± 3.1 (F), 13.9 ± 2.8 (M) **G1**: 13.2 ± 3.4 (F), 13.4 ± 2.7 (M)	**Con**: 68, 34 F **G1**: 72, 38 F	Quantum II, RJL; SF, 50 kHz; 4-electrode	**PhA**	PhA, **Con**: 6.2 ± 1.0, **G1**: 4.2 ± 0.8 (F)*, **Con**: 5.4 ± 0.8, **G1**: 4.4 ± 0.7 (M)*
**Hospitalization**							
Pileggi, 2016 [103]	Brazil	Extensive range of diagnoses	**Con**: 1 m-18y **G1**: 1 m-18y **Con1**: 5-18y **G1a**: 5-18y **Con2**: 5-18y **G1b**: 5-18y	**Con**: 210, N/A F **G1**: 237, N/A F **Con1**: 69 M **G1a**: 84 M **Con2**: 82 F **G1b**: 79 F	N/A	R	R, **Con**: 715.8 ± 92.1, **G1**: 736.1 ± 133.8*, **Con1**: 662.3 ± 97.0, **G1a**: 704.0 ± 123.5*, **Con2**: 744.9 ± 85.6, **G1b**: 746.3 ± 121.3*;
						Xc	Xc, **Con**: 65.3 ± 9.9, **G1**: 58.5 ± 14.1, **Con1**: 65.3 ± 8.4, **G1a**: 63.2 ± 10.1*, **Con2**: 70.2 ± 7.5, **G1b**: 63.3 ± 12.3*;
						**PhA**	PhA, **Con**: 5.3 ± 0.9, **G1**: 4.6 ± 1.2, **Con1**: 5.7 ± 0.8, **G1a**: 5.3 ± 1.1*, **Con2**: 5.5 ± 0.7; **G1b**: 4.9 ± 0.9*
**Kidney diseases**							
Brantlov, 2019 [104]	Denmark	Nephrotic syndrome	**Con**: 2-10y **G1**: 2-10y	**Con**: 38, 15 F **G1**: 8, 1 F	Hydra 4200, Xitron; MF; 4-electrode	R	R, **Con**: 721.9 ± 65.3, **G1**: 421.5 ± 44.7*;
						Xc	Xc, **Con**: 66.9 ± 8.1, **G1**: 22.1 ± 5.4*;
						**PhA**	PhA, **Con**: 5.3 ± 0.5, **G1**: 3.0 ± 0.6*
Varda, 2023 [119]	Slovenia	Chronic kidney disease, hypertension	**Con**: 14.0 ± 4.0y **G1**: Chronic kidney disease, 16.0 ± 6.0y **G2**: Hypertension, 15.0 ± 6.0y	**Con**: 31, 18 F **G1**: 37, 14 F **G2**: 46, 9 F	Nutrilab, Akern; SF, 50 kHz; 4-electrode	**PhA**	PhA, **Con**: 6.0 ± 0.0, **G1**: 6.7 ± 1.3*, **G2**: 7.0 ± 1.4*
**Metabolic diseases**							
Wiech, 2018 [115]	Poland	Type 1 diabetes	**Con**: 10.8 ± 3.7y **G1**: 10.8 ± 3.7y	**Con**: 63, 28 F **G1**: 63, 28 F	BIA 101, Akern; SF, 50 kHz; 4-electrode	R	R, **Con**: 660.0 ± 94.1, **G1**: 684.9 ± 99.3;
						Xc	Xc, **Con**: 57.62 ± 10.41, **G1**: 63.83 ± 6.93*
						**PhA**	PhA, **Con**: 4.85 ± 0.86, **G1**: 5.62 ± 0.81*
Nsamba, 2022 [114]	Uganda	Type 1 diabetes	**Con**: 6-17.9y **G1**: 6-17.9y	**Con**: 164, 85 F **G1**: 164, 81 F	DC-430MA, Tanita; SF, 50 kHz; 4-electrode	R	R, **Con**: 608.9 ± 86.9, **G1**: 585.6 ± 83.2;
						Xc	Xc, **Con**: 52.1 ± 9.7, **G1**: 54.6 ± 10.9;
						**PhA**	PhA, **Con**: 4.94 ± 0.81, **G1**: 5.32 ± 0.80*
**Neurological impairment**							
Calcaterra, 2019 [105]	Italy	Cerebral palsy, epileptic encephalopathy, and dysmorphic syndromes	**Con**: 15.0 ± 1.7y **G1**: 14.1 ± 5.3y	**Con**: 143, 16 F **G1**: 52, 21 F	BIOSMART, Eupraxia; MF; 4-electrode	RI	RI, **Con**: 315.5 ± 62.5, **G1**: 645.2 ± 114.4*;
						XcI	XcI, **Con**: 33.1 ± 5.9, **G1**:37.7 ± 11.8*;
						**PhA**	PhA, **Con**: 6.2 ± 0.9, **G1**: 3.2 ± 0.8*
**Nutritional diseases**							
Mika, 2004 [106]	Germany	Anorexia nervosa	**Con**: 15.1 ± 2.2y **G1**: 14.4 ± 1.5	**Con**: 19 F **G1**: 21 F	2000, Data Input; MF; 4-electrode	R	R, **Con**: 663 ± 62, **G1**: 752 ± 64*;
						Xc	Xc, **Con**: 65.4 ± 8.1, **G1**: 61.7 ± 9.5;
						**PhA**	PhA, **Con**: 5.6 ± 0.5, **G1**: 4.6 ± 0.4*
Guida, 2006 [118]	Italy	Overweight and obesity	**Con**: 8y **G1**: Overweight, 8y **G2**: Obesity, 8y	**Con**: 218, 109 F **G1**: 135, 78 F **G2**: 111, 52 F	Quantum 101, RJL; SF, 50 kHz; 4-electrode	R	R, **Con**: 730.4 ± 73.7, **G1**: 683.6 ± 59.2*, **G2**: 635.7 ± 72.1*;
						Xc	Xc, **Con**: 76.0 ± 13.8, **G1**: 72.4 ± 14.1*, **G2**: 70.4 ± 14.7*;
						**PhA**	PhA, **Con**: 6.0 ± 1.2, **G1**: 6.1 ± 1.3, **G2**: 6.4 ± 1.4*
Moreno, 2008 [107]	France	Anorexia nervosa	**Con**: 13.8 ± 3.4y **G1**: 15.2 ± 1.7y	**Con**: 17 F **G1**: 23 F	Hydra 4200, Xitron; MF; 4-electrode	**PhA**	PhA, **Con**: 6.4 ± 0.6, **G1**: 5.5 ± 1.0*
Bonaccorsi, 2009 [126]	Italy	Overweight and obesity	**Con1**: 8y **G1**: 8y **Con2**: 8y **G2**: 8y	**Con1**: 176 F **G1**: 34 F **Con2**: 183 M **G2**: 56 M	STA, Akern; SF, 50 kHz; 4-electrode	R	R, **Con1**: 768.9 ± 79.1, **G1**: 702.8 ± 69.1*, **Con2**: 731.3 ± 74.2, **G2**: 684.8 ± 67.9*;
						Xc	Xc, **Con1**: 84.4 ± 10.4, **G1**: 81.4 ± 6.7, **Con2**: 61.7 ± 7.2, **G2**: 57.4 ± 6.8*;
						**PhA**	PhA, **Con1**: 6.3 ± 0.6, **G1**: 6.6 ± 0.3*, **Con2**: 6.4 ± 0.6, **G2**: 6.4 ± 0.6
Barufaldi, 2011 [116]	Brazil	Obesity	**All**: 10.8 ± 2.9y **Con1**: Children, N/A **G1**: Children, N/A **Con2**: Adolescents, N/A **G2**: Adolescents, N/A	**Con1**: 1302, N/A F **G1**: 79, N/A F **Con2**: 1701, N/A F **G2**: 122, N/A F	Quantum II, RJL; SF, 50 kHz; 4-electrode	R	R, **Con1**: 691.9 ± 64.2, **G1**: 633.9 ± 53.9*, **Con2**: 612.3 ± 81.1, **G2**: 558.5 ± 67.3*;
						Xc	Xc, **Con1**: 66.7 ± 8.4; **G1**: 62.5 ± 5.9*, **Con2**: 65.1 ± 8.1, **G2**: 60.9 ± 7.6*;
						**PhA**	PhA, **Con1**: 5.5 ± 0.6, **G1**: 5.7 ± 0.5*, **Con2**: 6.1 ± 0.8, **G2**: 6.3 ± 0.7*
Girma, 2018 [113]	Ethiopia	Severe acute malnutrition	**Con**: 28 ± 15 m **G1**: 36 ± 24 m	**Con**: 80, 42 F **G1**: 55, 19 F	Quadscan 4000, Bodystat; MF; 4-electrode	R	R, **Con**: 839 ± 118, **G1**: 825 ± 270;
						Xc	Xc, **Con**: 57 ± 11, **G1**: 33 ± 17*;
						**PhA**	PhA, **Con**: 3.8 ± 0.7, **G1**: 2.2 ± 0.7*
Oliveira Filho, 2019 [120]	Brazil	Obesity	**Con**: 16.0 ± 1.0y **G1**: 16.2 ± 0.9y	**Con**: 411, 157 F **G1**: 78, 32 F	Quantum II, RJL; SF, 50 kHz; 4-electrode	R	R, **Con**: 602.1 ± 91.3, **G1**: 512.8 ± 78.6*;
						Xc	Xc, **Con**: 68.3 ± 8.3, **G1**: 61.1 ± 7.3*;
						**PhA**	PhA, **Con**: 6.5 ± 0.8, **G1**: 6.9 ± 0.9*
Girma, 2021 [108]	Ethiopia	Severe acute malnutrition	**Con**: 38 m **G1**: 29–36 m	**Con**: 120, 60 F **G1**: 350, 152 F	Quadscan 4000, Bodystat; MF; 4-electrode	R	R, **Con**: 826 ± 109, **G1**: 888 ± 252;
						Xc	Xc, **Con**: 62 ± 13, **G1**: 37 ± 16;
						**PhA**	PhA, **Con**: 4.3 ± 1.0, **G1**: 2.5 ± 1.1
Nagano, 2020 [112]	Japan	Acute malnutrition	**Con**:3.4 ± 3.1y **G1**: 2.6 ± 2.6y	**Con**: 71, 28 F **G1**: 10, 0 F	N/A, RJL; SF, 50 kHz; 4-electrode	**PhA**	PhA, **Con**: N/A, **G1**: 2.4 ± 0.7
Popiołek, 2020 [109])	Poland	Anorexia nervosa	**Con**: 17.5 ± 2.0y **G1**: 17.0 ± 3.0y	**Con**: 42 F **G1**: 42 F	SFB7, ImpediMed; MF; 4-electrode	R	R, **Con**: 616.6 ± 48.5, **G1**: 678.2 ± 88.0*;
						Xc	Xc, **Con**: 60.4 ± 6.6, **G1**: 49.1 ± 16.4*;
						**PhA**	PhA, **Con**: 5.5, **G1**: 4.3*;
						Z	Z, **Con**: 619.6 ± 48.6, **G1**: 680.2 ± 88.0*
Almeida, 2021 [110]	Brazil	Malnutrition, overweight, obesity	**Con**: N/A; **G1**: Malnutrition, N/A **G2**: Overweight, N/A **G3**: Obesity, N/A	**Con**: 93, N/A F **G1**: 4, N/A F **G2**: 15, N/A F **G3**: 8, N/A F	450, Biodynamics; SF, 50 kHz; 4-electrode	R	R, **Con**: 688.9 ± 95.9, **G1**: 806.5 ± 85.6, **G2**: 624.4 ± 95.0, **G3**: 594.1 ± 70.1*;
						Xc	Xc, **Con**: 78.8 ± 11.2, **G1**: 87.2 ± 14.9, **G2**: 73.0 ± 14.4, **G3**: 68.8 ± 9.1*;
						**PhA**	PhA, **Con**: 6.6 ± 1.0, **G1**: 6.2 ± 1.0, **G2**: 6.8 ± 1.3, **G3**: 6.7 ± 0.8*
Howe, 2021 [127]	USA	Overweight and obesity	**Con**: 11.8 ± 3.0y **G1**: 11.1 ± 2.9y **G2**: 10.4 ± 2.7y	**Con**: 37, N/A F **G1**: 10, N/A F **G2**: 11, N/A F	770, InBody; MF; 8-electrode	**PhA**	PhA, **Con**: 5.4 ± 0.1, **G1**: 5.4 ± 0.2, **G2**: 5.4 ± 0.2
Macena, 2021 [121]	Brazil	Acute malnutrition – Stunting	**Con**: 36.9 ± 9.4y **G1**: At risk of stunting, 43.8 ± 8.5y **G2**: Stunted, 36.3 ± 8.9y	**Con**: 25, 9 F **G1**: 38, 22 F **G2**: 37, 9 F	BI1010, Sanny; SF, 50 kHz; 4-electrode	R	R, **Con**: 775.0, **G1**: 823.7, **G2**:793.4;
						Xc	Xc, **Con**: 53.9, **G1**: 65.2*, **G2**: 63.4*;
						**PhA**	PhA, **Con**: 3.9, **G1**: 4.5*, **G2**: 4.6*
**Oncologic diseases**							
Tseytlin, 2010 [111]	Russia	Acute lymphoblastic leukemia	**Con**: 10.7 ± 3.4y (F), 10.5 ± 3.5y (M) **G1**: 10.7 ± 3.4y (F), 10.5 ± 3.5y (M)	**Con**: 220, 108 F **G1**: 220; 108 F	ABC-01, Medas; SF, 50 kHz; 4-electrode	R	R, **Con**: 678 ± 84, **G1**: 715 ± 86 (F)*, **Con**: 626 ± 99, **G1**: 677 ± 79 (M)*;
						Xc	Xc, **Con**: 74.7 ± 9.4, **G1**: 65.3 ± 7.5 (F)*, **Con**: 69.6 ± 10.0, **G1**: 62.3 ± 7.0 (M)*;
						**PhA**	PhA, **Con**: 6.3 ± 0.7, **G1**: 5.4 ± 0.6 (F)*, **Con**: 6.4 ± 0.7, **G1**: 5.4 ± 0.4 (M)*
**Respiratory diseases**							
Hauschild, 2016 [123]	Brazil	Cystic fybrosis	**Con**: 8.8y with 7.1-11.4y IQR^†^ **G1**: 8.5y with 7.6-10.8y IQR^†^	**Con**: 24, 10 F **G1**: 46, 22 F	310, Biodynamics; SF, 50 kHz; 4-electrode	RI	RI, **Con**: 25.1, **G1**: 20.3*;
						**PhA**	PhA, **Con**: 5.0, **G1**: 5.0

Abbreviations: BIA, bioelectrical impedance analysis; CDK, chronic kidney disease; Con, control group; F, females; IQR, interquartile range; m, months; M, males; m, months; MF, multi-frequency bioelectrical impedance device; N/A, not available; PhA, Phase angle (º); R, resistance (Ω); RI, resistance index (R/cm; R/m); SF, single frequency bioelectrical impedance device (50 kHz); sPhA, standard Phase angle; Xc, reactance (Ω); XcI, reactance index (Xc/cm; Xc/m); y, years; Z, impedance

* Significant differences between pediatric patients and health age-matched control groups at α = 0.05 level

† Age and raw BIA values presented as median and interquartile range

‡ Although chronic kidney disease is primarily a kidney condition, it is included in cardiovascular disease analysis as presented in group analysis by Švigelj et al. (2021)

## Scientific remarks and future recommendations

Although this review provides a global perspective on how PhA is expected to be modulated by body composition in youth, attention must be given to relevant scientific remarks. Beyond the segmented view of how body components at each level may impact the dielectric properties of the body and, consequently, PhA, shifting the paradigm from a reductionist approach to an integrative framework is essential for addressing the network of biological interactions (i.e., global network dynamics) [129]. Despite levels themselves possessing unique properties that should not be confused with one another [11], the structural and functional connectivity of physiological networks underlying individual body composition levels needs further attention in the field of BIA. Currently, there is a promising application of BIA in the field of body composition, as PhA and other raw parameters possess associations with interdependent body components at multiple levels, possibly reflecting inherent characteristics (e.g., ICW at the molecular level; BCM at the cellular level; muscle tissue at the tissue level). However, further investigations exploring simultaneously all levels of body composition analysis (i.e., ecological approach) are needed to improve the mechanistic interpretation of PhA. This will contribute to understanding how dielectric properties are determined in each body component at different levels and, successively at higher levels, highlighting which component or combinations of components exert the greatest influence on PhA.

Another remark is that several methodological factors must be considered when interpreting dielectric data, particularly from in vitro studies, as key experimental conditions can significantly influence measured capacitance and conductivity. Differences in experimental models, sample size, bath electrolytes’ composition, buffers, and temperature, as well as in the characteristics of the equipment used (i.e., frequency, current intensity, electrode placement, and configuration) impact dielectric properties, potentially limiting the direct applicability of findings to raw BIA variables. Furthermore, the extrapolation of isolated conductivity and capacitance to whole-body PhA remains challenging, as systemic regulatory mechanisms, metabolic activity, and fluid shifts at multiple levels of body composition cannot be replicated in controlled laboratory conditions. To enhance the reproducibility and physiological relevance of dielectric measurements explaining PhA, future research must establish standardized protocols, report key experimental conditions, and integrate comparative in vitro and in vivo research. Furthermore, integrating multifrequency analysis may improve the diagnostic ability of PhA, given the frequency-dependent nature of the conductivity and permittivity of body components [68]. By addressing these limitations, a more accurate understanding of how PhA reflects physiological components in youth can be achieved, strengthening its role in body composition assessment and health monitoring.

When looking at the available evidence exploring the biological intricacies of raw BIA variables in human models, additional reflections must be expressed to guide future investigations. The upcoming investigations should prioritize validated gold-standard reference methods to assess the multilevel components of youth. This will enhance the reliability and reproducibility of results and provide more accurate relationships with PhA. In addition, there is a need to develop longitudinal research exploring the biophysical impact resulting from changes across multilevel determinants of body composition, being of most interest in periods of profound physiological and morphological changes.

Future reflections must also be made on the practical application of PhA. While previous research has demonstrated that this raw BIA parameter can inform about multiple parameters of health in youth [8, 52, 60], it is not expected to offer similar levels of precision in body composition and physical performance as gold-standard techniques (e.g., magnetic resonance imaging and computed tomography for body composition measurement; maximum oxygen consumption dynamometry test for physical function assessment). Similarly, for metabolic health assessment, traditional biochemical markers (e.g., circulating hormones, lipids, and signaling proteins) provide more reliable information than PhA [52]. These findings stem from the fact that PhA is an indirect marker of the aforementioned domains, primarily reflecting cell membrane integrity and fluid distribution [1]. Even so, its non-invasive, cost-effective, and easily measurable nature makes this parameter particularly attractive in multiple settings in youth, including pediatric physical health assessments for early detection of disease signs or unfavorable growth patterns and training progression monitoring in young athletes. On a large scale (e.g., schools, public health programs, and resource-limited settings), the PhA can also serve as a valid marker reflecting health-related information and giving support to health promotion policies.

In addition, further research using localized BIA is required to evaluate the precision and applicability of regional PhA in reflecting site-specific and enhancing the monitoring of whole-body components. This is of particular interest in contexts where specific systemic health conditions (e.g., obesity) modulate the associations between whole-body BIA and body components, potentially reducing the explanatory power of whole-body raw BIA parameters. In this field, the usefulness of localized BIA was recently demonstrated, with arm PhA measured at 5 and 15 kHz capturing distinct magnitudes and direction associations with bone-related outcomes, depending on body weight status [130]. Lastly, we challenge upcoming researchers to dive into biophysical measurements and investigate whether multilevel body composition variables are associated with biophysical parameters of other disregarded biophysical models in pediatric populations (e.g., parallel models).

## Data Availability

No datasets were generated or analysed during the current study.

## Abbreviations

BCM: Body cell mass
BIA: Bioelectrical impedance analysis
BLM: Bilayer lipid membrane
CSA: Cross-sectional area
ECW: Extracellular water
ECW:ICW: Extracellular-to-intracellular water ratio
FA: Fatty acids
FFM: Fat-free mass
FM: Fat mass
ICW: Intracellular water
PhA: Phase angle
R: Resistance
SMM: Skeletal muscle mass
TBW: Total body water
X: Reactance
Xc: Capacitive reactance
Z: Impedance
